# Image Quality in Children with Low-Radiation Chest CT Using Adaptive Statistical Iterative Reconstruction and Model-Based Iterative Reconstruction

**DOI:** 10.1371/journal.pone.0096045

**Published:** 2014-05-14

**Authors:** Jihang Sun, Yun Peng, Xiaomin Duan, Tong Yu, Qifeng Zhang, Yong Liu, Di Hu

**Affiliations:** Imaging Center, Beijing Children's Hospital, Capital Medical University, Beijing, China; Mayo Clinic College of Medicine, United States of America

## Abstract

**Objective:**

To evaluate noise reduction and image quality improvement in low-radiation dose chest CT images in children using adaptive statistical iterative reconstruction (ASIR) and a full model-based iterative reconstruction (MBIR) algorithm.

**Methods:**

Forty-five children (age ranging from 28 days to 6 years, median of 1.8 years) who received low-dose chest CT scans were included. Age-dependent noise index (NI) was used for acquisition. Images were retrospectively reconstructed using three methods: MBIR, 60% of ASIR and 40% of conventional filtered back-projection (FBP), and FBP. The subjective quality of the images was independently evaluated by two radiologists. Objective noises in the left ventricle (LV), muscle, fat, descending aorta and lung field at the layer with the largest cross-section area of LV were measured, with the region of interest about one fourth to half of the area of descending aorta. Optimized signal-to-noise ratio (SNR) was calculated.

**Result:**

In terms of subjective quality, MBIR images were significantly better than ASIR and FBP in image noise and visibility of tiny structures, but blurred edges were observed. In terms of objective noise, MBIR and ASIR reconstruction decreased the image noise by 55.2% and 31.8%, respectively, for LV compared with FBP. Similarly, MBIR and ASIR reconstruction increased the SNR by 124.0% and 46.2%, respectively, compared with FBP.

**Conclusion:**

Compared with FBP and ASIR, overall image quality and noise reduction were significantly improved by MBIR. MBIR image could reconstruct eligible chest CT images in children with lower radiation dose.

## Introduction

Several techniques, like automatic adjustment of tube current [1], reduced tube voltage [2], noise reduction filters [3] and a higher pitch [4] allowed radiologists to effectively diagnose diseases using lower radiation doses during computed tomography (CT) scans. However, these techniques are limited by an increased noise and a degraded image quality when the radiation dose is too low, mostly due to the reconstruction algorithm used in most CT systems. Indeed, the filtered back-projection (FBP) technique is the traditional way to reconstruct images from multi-angle images. It was developed more than 25 years ago [5] and is greatly limited from further reducing the radiation dose. Thus, FBP increases the noise at low radiation doses because its algorithm considers images from each angle as being fully free of noise and equally valid [1], [6].

Adaptive statistical iterative reconstruction (ASIR) is a new CT reconstruction algorithm using statistical models to reduce image noise and producing better image quality [7], [8]. ASIR also allow further reduction of radiation dose by 32–65% [8]. Subsequent phantom and patients studies showed that ASIR provides images that are suitable for diagnosis using low radiation doses [9]–[13].

The recently model-based iterative reconstruction (MBIR) algorithm is a new iterative reconstruction technique that is much more complex and advanced than ASIR [7], [14]–[17]. MIBR significantly reduces artifacts, improves spatial resolution and improves image quality [7] in colon [16], abdominal [17] and chest [15] imaging. However, few data are available on its image quality and noise in specific populations.

In the present study, MBIR, ASIR, and FBP were used to reconstruct CT images from children. MIBR-reconstructed images quality and noise were evaluated, and compared with the same images reconstructed using 60% ASIR and conventional FBP to evaluate the applicability of MBIR in reconstructing low-dose chest CT scans in children.

## Subjects and Methods

The study protocol was approved by the Ethics Committees of Beijing Children's Hospital (Beijing, China), and the parents provided a written consent for their children.

### Patients

Forty-five children who underwent enhanced chest CT scans at the Beijing Children's Hospital (Beijing, China) between March 11^th^, 2012 and June 17^th^, 2012 were included. Children were divided into three groups: young infants (0–12 months of age), older infants (1–2 years of age) and preschoolers (3–6 years of age).

### Instruments and devices

All included children were scanned using a Discovery 750 scanner (GE Healthcare, Waukesha, WI, USA) with a tube voltage of 120 KV, pitch of 1.375, and rotation speed of 0.8 seconds. Automatic tube current modulation (ATCM) was used to modulate the tube current, which was set between 10 and 350 mA. An age-dependent noise index (NI) was used for acquisition: NI = 12 for 0–12 months of age, NI = 15 for 1–2 years of age, and NI = 17 for 3–6 years of age. Images were retrospectively reconstructed into three series with a 5-mm slice thickness: series A using MBIR, series B using 60% of ASIR and 40% of conventional FBP, and series C using FBP. Since the children mostly refused to cooperate, CT scans were performed when the children were asleep or sedated with oral intake of 10% chloral hydrate (0.5 ml/Kg) half an hour before scanning. The scan covered the area between the entrance to the chest and the base of the lung.

### Subjective assessment of the image quality

All images were transferred to a GE AW4.5 CT workstation (GE Healthcare, Waukesha, WI, USA). Two experienced radiologists (one with an 8-year experience in pediatric radiography who majored in chest X-ray and CT, and the other with a 3-year experience in adult radiography and a 2-year experience in pediatric radiography who majored in chest X-ray and CT) were asked to independently evaluate images' quality. The radiologists were allowed to adjust the window width and window level to their usual condition.

Image quality was evaluated from the following aspects [10], [18]: subjective noise in mediastinal window and lung window using a 5-point scale (5 =  rare noise; 4 =  little noise; 3 =  acceptable noise; 2 =  worse than acceptable noise; and 1 =  unacceptable noise); visibility of tiny structures (including small airways contrast), such as pulmonary vessels, mediastinal tissues, lymph nodes, and lesions in mediastinal window and lung window using a 5-point scale (5 =  structures are displayed clearly with excellent contrast; 4 =  good contrast; 3 =  displayed structures are not clear, but enough for diagnosis; 2 =  displayed structures are not clear and could not be used for diagnosis; and 1 =  very hard to distinguish the fine structures); the clarity of the edges of normal anatomic structures and lesions including fat-muscle boundary using a 5-point scale (5 =  clearly displayed with sharp boundary; 4 =  clearly displayed boundary; 3 =  the boundary was not very clear but could be used for diagnosis; 2 =  blurred boundary and could not be used for diagnosis; and 1 =  could not display the boundary); and number of lesions (lesions <1 cm and >1 cm were counted separately; invasive and diffuse pulmonary lesions were counted per pulmonary segment; single-side or encapsulated pleural effusion was regarded as one lesion). Overall diagnostic confidence was evaluated with a 5-point scale (5 =  excellent confidence; 4 =  good confidence; 3 =  insufficient confidence but not affect diagnosis; 2 =  insufficient confidence and could not establish the diagnosis; and 1 =  without confidence).

### Evaluation of the objective noise

A circular region of interest (20–120 mm^2^) in the image with the largest cross-section area of the left ventricle (LV) was selected by the two radiologists. Then, CT density was measured and the standard deviation (SD) was calculated. CT densities of the back muscles, subcutaneous fat, descending aorta, and the pulmonary fields without lesion or obvious lung markings in the same region of the same layer were also measured, and the SDs were calculated. The objective image noise of the tissue was calculated as the average SD, and then the signal-to-noise ratio (SNR) of each tissue was calculated using the following formula: SNR =  CT density/objective noise. The area of the interest region was generally about half of the area of the cross-section area of the descending aorta at the image of the same layer. However, as some children had thin muscles and adipose tissues, the region of interest was about one fourth of the area of the cross-section area of the descending aorta, and the shape of the region could be changed in the evaluation.

### Radiation dose

Parameters of the X-ray radiation dose included CT dose index of volume (CTDIvol) and dose length product (DLP). Effective dose (ED) was calculated using the following formula: ED =  DLP×W (W: conversion factors of different parts of the body. The W is 0.017 for the chest, according to the European guidelines on quality criteria for computed tomography [19]).

### Statistical analysis

Objective noise, subjective quality evaluation, and radiation dosage were recorded in details. Mean±SD was calculated for subjective quality evaluations and objective noise. Differences between MBIR, ASIR, and FBP images were compared using ANOVA, and a Student's paired t-test was used to determine the significance of differences between image pairs. To account fot multiple statistical, a Bonferroni correction was applied, and significance was assumed only when the P-value was <0.016. Signal-to-noise ratio was calculated and compared among the groups. The noise was compared among the age-groups. Kappa statistics were used to evaluate the consistency between the two radiologists' diagnoses. All statistical analyses were performed using SPSS17.0 (SPSS Inc., Chicago, IL, USA).

## Results

### Patients' characteristics

Forty-five children (25 boys and 20 girls) were included in the present study. The median age was 1.8 years (ranging from 28 days to 6 years). Among these, 41 underwent CT scan for pneumonia (including 9 children with necrotizing pneumonia), one for a neurogenic tumor, one for a teratoma, one for foregut malformation, and one without any lesion (this child was initially suspected with anterior mediastinum mass, which was later proved to be normal thymus tissue). All 178 lesions, including 122 parenchymal infiltrative lesions (including small airway wall thickening, pulmonary atelectasis, and necrotizing pneumonia), 17 pleural lesions, 36 pulmonary emphysema, and three pulmonary space-occupying lesions, were displayed in each series.

### Subjective image quality evaluation

The subjective image quality evaluations of the three series are displayed in Table 1. MBIR images showed the best subjective noise with significantly lesser granular noise artifacts. However, MBIR images displayed slightly blurred boundaries.

**Table 1:** Subjective quality evaluation of the images of the three series.

Image reconstruction method	Number of lesions		Image noise		Visibility of tiny structures		Clarity of the lesion edges	Diagnostic confidence
	<1 cm	>1 cm	Mediastinal window	Lung window	Mediastinal window	Lung window		
	Overall							
Series A	115	63	4.9±0.3* #	4.9±0.3* #	5.0±0.0* #	5.0±0.0* #	4.1±0.3* #	5.0±0.0* #
Series B	115	63	3.9±0.3*	4.3±0.5*	3.9±0.3*	3.9±0.3*	4.6±0.5*	5.0±0.0*
Series C	115	63	3.3±0.5#	3.0±0.2#	3.2±0.6#	3.3±0.5#	5.0±0.1#	5.0±0.0#
	Young infants (0–12 months of age), NI = 12							
Series A	46	22	4.9±0.2* #	5.0±0.0* #	5.0±0.0* #	5.0±0.0* #	4.0±0.0* #	5.0±0.0* #
Series B	46	22	3.9±0.2*	4.7±0.5*	3.9±0.3*	4.0±0.0*	4.6±0.2*	5.0±0.0*
Series C	46	22	3.7±0.5#	3.0±0.0#	3.1±0.5#	3.1±0.2#	4.9±0.2#	5.0±0.0#
	Older infants (1–2 years of age), NI = 15							
Series A	32	22	4.9±0.3* #	4.9±0.3* #	5.0±0.0* #	5.0±0.0* #	4.0±0.0* #	5.0±0.0* #
Series B	32	22	4.0±0.4*	4.0±0.0*	3.8±0.5*	3.8±0.4*	4.5±0.4*	5.0±0.0*
Series C	32	22	3.2±0.4#	3.1±0.3#	3.2±0.4#	3.1±0.3#	5.0±0.0#	5.0±0.0#
	Preschoolers (3–6 years of age), NI = 17							
Series A	37	19	4.9±0.3* #	4.9±0.3* #	5.0±0.0* #	5.0±0.0* #	4.2±0.4* #	5.0±0.0* #
Series B	37	19	4.0±0.0*	3.7±0.5*	3.9±0.3*	3.8±0.4*	4.7±0.3*	5.0±0.0*
Series C	37	19	3.0±0.0#	3.0±0.0#	3.2±0.3#	3.9±0.3#	5.0±0.0#	5.0±0.0#

NI: Noise index; series A: MBIR; series B: 60% of ASIR and 40% of conventional FBP; series C: FBP.

*P<0.01 Series A vs. Series B,

# *P*<0.01 Series A vs. Series C.

### Objective image quality

Table 2 displays the objective image noise and SNR. Results were from the objective image noise of the LV. There were significant differences in the objective noise value among MBIR, ASIR and FBP images (P<0.01). The objective noise for MBIR was reduced by 30.2% and 51.9%, compared with ASIR and FBP, respectively (*P*<0.01). There were significant differences in SNR among the three groups (*P*<0.01). CT image quality for MBIR was improved by 44.4% and 108.4%, compared with ASIR and FBP, respectively (*P*<0.01).

**Table 2:** Noise, SNR, and CNR in the images of the three series.

	Left ventricle noise	Muscles noise	Fat tissue noise	Aorta noise	Lung noise	SNR left ventricle	SNR muscles	SNR Fat tissue	SNR aorta	SNR lung
	Overall									
Series A	5.97±1.06* #	5.94±1.32* #	8.36±2.35 * #	8.79±2.62* #	14.56±5.08* #	6.45±1.82* #	9.05±2.00* #	13.35±4.73* #	5.13±3.09* #	54.21±18.53* #
Series B	9.10±1.62*	9.48±2.22*	12.81±3.73*	13.17±3.55*	20.34±6.32*	4.21±1.43*	5.41±1.35*	8.97±4.09*	3.46±2.22*	37.73±15.54*
Series C	13.34±2.44#	13.43±2.91#	15.95±4.07#	16.43±3.63#	24.53±7.63#	2.88±0.99#	3.81±0.85#	7.11±2.93#	2.69±1.72#	31.10±12.05#
	Young infants (0–12 months of age), NI = 12									
Series A	5.29±0.88* #	5.14±0.86* #	7.23±1.94* #	7.43±2.78* #	16.00±6.74* #	7.12±2.25* #	10.74±2.13* #	15.82±4.55* #	5.98±3.54* #	48.99±23.00* #
Series B	7.58±1.20*	8.28±1.52*	10.56±2.67*	12.31±3.56*	20.27±7.56*	5.10±1.82*	6.04±1.45*	10.82±4.34*	3.92±2.40*	36.65±14.79*
Series C	10.92±1.48#	11.26±1.59#	12.57±2.70#	14.73±3.37#	23.41±8.11#	3.54±1.27#	4.40±0.84#	8.92±3.18#	3.18±1.89#	31.29±13.04#
	Older infants (1–2 years of age), NI = 15									
Series A	5.92±0.96* #	6.22±1.06* #	8.88±2.16* #	8.98±2.31* #	14.98±3.94* #	6.02±1.39* #	7.83±1.03* #	12.45±4.76* #	4.72±2.36* #	49.86±12.64* #
Series B	10.12±1.20*	9.93±2.37*	12.85±3.31*	12.70±4.00*	22.43±5.58*	3.50±0.65*	4.93±1.26*	8.95±4.10*	3.37±1.83*	32.72±9.70*
Series C	14.62±1.59#	13.87±2.72#	15.82±2.79#	15.75±3.27#	27.72±8.49#	2.43±0.45#	3.49±0.73#	7.03±2.43#	2.53±1.25#	27.02±9.58#
	Preschoolers (3–6 years of age), NI = 17									
Series A	6.68±0.90* #	6.56±1.37* #	9.13±2.56* #	10.01±2.43* #	12.84±3.62* #	6.07±1.43* #	8.17±1.67* #	11.47±3.76* #	4.57±3.20* #	62.33±17.25* #
Series B	9.93±1.37*	10.38±1.76*	15.03±4.13*	14.33±2.84*	19.02±5.26*	3.78±1.15*	5.11±0.94*	7.12±3.04*	3.05±2.40*	42.15±16.28*
Series C	14.91±1.91#	15.31±2.25#	19.41±3.96#	18.58±2.96#	23.51±5.76#	2.52±0.72#	3.45±0.61#	5.36±1.80#	2.30±1.89#	33.62±9.83#

SNR: signal-to-noise ratio; series A: MBIR; series B: 60% of ASIR and 40% of conventional FBP; series C: FBP.

*P<0.01 Series A vs. Series B,

# *P*<0.01 Series A vs. Series C.

The objective noise value differed significantly between the age groups (*P*<0.01). The noise in the newborn group was reduced by 15.5% and 19.2%, compared with the infant and the preschool children groups, respectively (*P*<0.01), and no significant difference was detected between the infant and the preschool children groups (*P* = 0.37).

### Inter-observer consistency and radiation dose

The inter-observer *Kappa* value was 0.89, showing a good consistency. The subjects had a mean CTDIvol of 0.67±0.17 mGy, a mean DLP of 14.7±3.1 mGy and a mean radiation dose of 0.25±0.05 mSv.

## Discussion

The traditional FBP technique provides limited image quality when using low radiation dose CT scanning because of the limits of its mathematical model. More recent reconstruction techniques, such as ASIR and MIBR, use complex statistical models to reduce noise and to improve image quality, even in a low-radiation dose setting [13], [20]–[22]. However, there is a lack of data in specific populations, especially MIBR in a pediatric population. Therefore, the aim of the present study was to compare MIBR, 60% ASIR and FBG in children who underwent CT.

**Figure 1:**
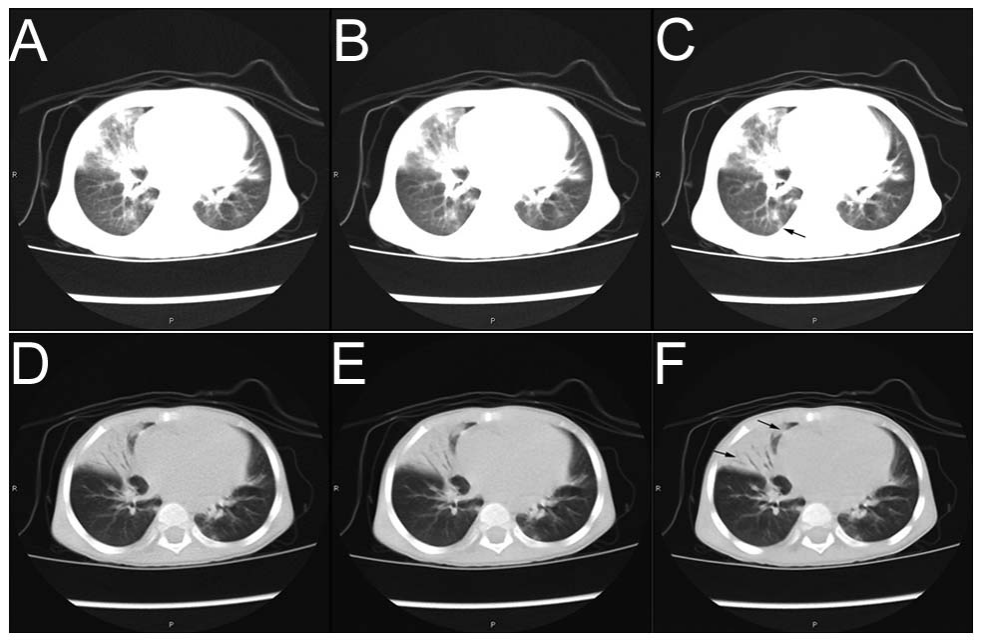
A 2-year old girl with fever for 15 days and cough for 2 days. X-ray suggested severe pneumonia, and enhanced CT scanning was performed for further examination. Tube voltage was 120–13 mA. CT revealed large areas of consolidation and air bronchogram, and the girl was diagnosed with adenovirus pneumonia. CT images displayed here were reconstructed with FBP (1-A), ASIR (1-B), and MBIR (1-C). The tube current of the presented layer was 13 mA, the window level was −700, and the window width was 1000. The MBIR image had the lowest noise in the three images, which displays the lung field and the region of the lesions clearly. Arrow shows the lesion is clearly displayed. Images were reconstructed with FBP (1-D), ASIR (1-E), and MBIR (1-F). The tube current of the presented layer was 13 mA, the window level was -450, and the window width was 1500. The MBIR image had the lowest noise among all three images. The lower arrow shows where that the air bronchogram is clearly displayed, while the upper arrow shows the air bronchogram that was not displayed in the other two images

**Figure 2:**
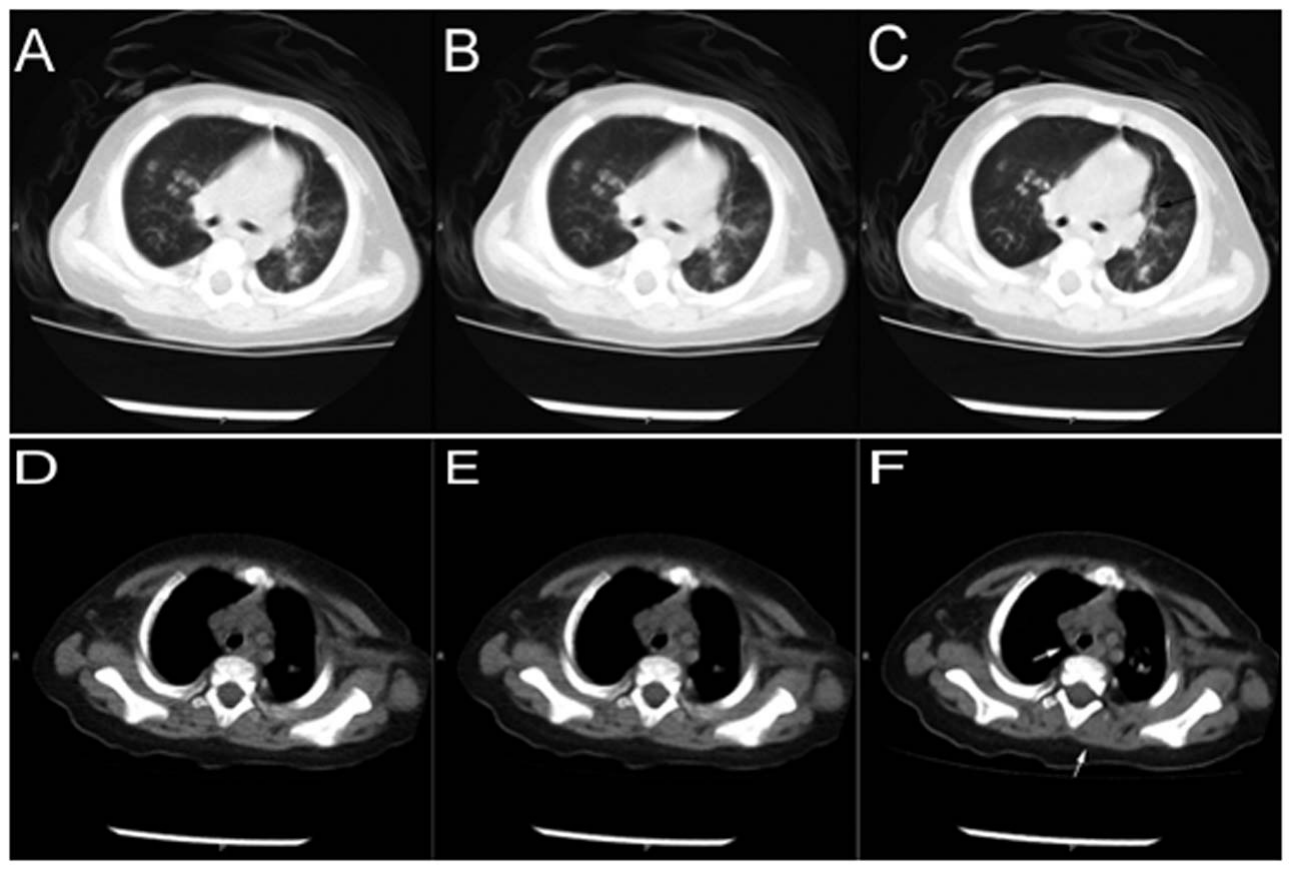
A 7-month old boy with repeated cough for 2 month was diagnosed with allergic invasive pulmonary aspergillosis (AIPA). Images were reconstructed with FBP (2-A), ASIR (2-B), and MBIR (2-C). The current of the presented layer was 22 mA, the window level was −700, and the window width was 1000. The scanning voltage was 120 kV, NI was 12, and the current was 22–24 mA. Images show multiple nodular high-density lesions in both lung fields, within which cysts could be found. Patchy shadows and multiple bronchiectasis are shown in the bilateral inferior lobes of the lung. The lowest noise was found in the MBIR image, which also displays the lung field and the region of the lesions clearly. The arrow shows the double track sign that is not displayed clearly in the other two images. Images were reconstructed with FBP (2-D), ASIR (2-E), and MBIR (2-F). The current of the presented layer was 22 mA, the window level was 40, and the window width was 400. The lowest noise was found in the MBIR image, which also displays the lung field and the region of the lesions clearly. The lower arrow shows the blurred margin of the muscle, while the upper arrow shows the lymph nodes behind the superior vena cava, which are not displayed clearly in the other two images.

In terms of subjective quality, MBIR images were significantly better than ASIR and FBP in image noise and visibility of tiny structures (Figure 1), but blurred edges were observed (Figure 2). In terms of objective noise, MBIR and ASIR reconstruction decreased the image noise by 55.2% and 31.8%, respectively, for LV compared with FBP. Similarly, MBIR and ASIR reconstruction increased the SNR by 124.0% and 46.2%, respectively, compared with FBP.

In the present study, all included children were between 0 and 6 years old, and the distribution of body fat and length were different from those of adults. Therefore, the association between the radiation dose and BMI was not clear in children. Consequently, we simply divided the children into different groups according to their age instead of their body weight or BMI. NI is associated with image noise, and higher image noise means that a higher NI is required, which is an approach that was previously reported, although with different NI [18]. In our hospital, the NI is commonly set at a higher value for younger patients, such as the NI is 2 points higher for infants and preschoolers. This allows for a better evaluation of the noise-decreasing ability of the MBIR technique. However, according to our experience, infants have several characteristics, including less pulmonary gas content, thin adipose tissue layer, and poor contrast of soft tissues; thus, a too high NI may affect diagnosis.

In the present study, only the NI in young infants was set 1 point higher than recommended by the manufacturer [23]. The NIs used in the present study were: 12 for young infants (0 to 12 months of the ages), 15 for older infants (1 to 2 years of the ages), and 17 for preschoolers (3 to 6 years of the ages). Because we were not sure whether higher NI will affect image quality when reconstructed by MBIR technique, only the data of plain CT scanning during the enhanced chest CT scanning was selected for each child for analysis, while NI was still used for enhanced CT scan. Plain CT scanning are mainly used for identifying calcification and the extent of the lesions; for some children, these images could not be used for diagnosis, but no adverse outcome (such as misdiagnosis) would occur because we could use the images of enhanced CT scan to diagnose the disease accurately. Evaluation of image quality is more important for plain CT scanning with higher NI and image noise.

Several researches demonstrated that ASIR can effectively decrease image noise, but blurred edges will also subsequently occur [7]–[13]. A previous study using 50% ASIR showed a noise reduction of 45.5% [15], while the present study showed a noise reduction of 31.8% using 60% ASIR. In the present study, we simultaneously evaluated image noise, tiny structures, and lesions' edge. According to findings from previous studies and our experience, 60% of the images reconstructed using ASIR had significantly decreased image noise and improved image quality in spite of several blurring artifacts, of which the influence was almost negligible. Thus, this is why 60% was selected as the reconstruction weight for series B.

The findings of the present study demonstrated that the subjective noise was significantly lower in children's MBIR images compared with FBP or ASIR images. Similarly, images in series A seemed more delicate, with less granular noise artifacts and more homogenous image density compared with series B and C, which concur with previous studies [8], [10]–[13]. The MBIR images could also display tiny structures, including the small bronchial walls, pleura, and small lymph nodes more clearly than the other two techniques (Figure 1,2), significantly facilitating diagnosis. In a study performed by Singh et al., blurred edges were found in the images reconstructed by ASIR [10]. Interestingly, these blurred edges were also found in MIBR, and were more serious in the images reconstructed by MBIR than by ASIR. In the present study, blurred edges were found for almost all the tissues with large density difference, but because the density inside these tissues was relatively homogeneous and because image noise was very low, these blurred edges did not significantly influence diagnosis. Studies revealed that most radiologists were not prone to diagnose using MBIR images [14]. However, both radiologists participating in the present study had reviewed IR images for more than one year, and they could readily use them for diagnosis. A score of 5 in diagnostic confidence was provided by both radiologists involved in the present study. However, a major limitation of MIBR is the long reconstruction time required to obtain the images, preventing us to use the high resolution mode. Therefore, the use of MIBR could be limited for some diagnoses, such as interstitial lung diseases.

In the present study, ATCM was used to modulate tube current, and tube currents in different layers were different. However, we chose the region of interest in the same layer to avoid the impact of different tube currents. On the other hand, the body type of children display greater changes with age than adults. In order to obtain a similar proportion of region of interest area in children with different body type, a circular region with the area about half of the cross-section of the aorta was chosen as the region of interest. Thus, the proportion of the area of the region of interest was the same in different individuals. In addition, the size of the region of interest could be modulated to facilitate the evaluation. However, as some children had thin muscles and adipose tissues due to disease or malnutrition, a region of interest of about one fourth of the area of the cross-section area of the descending aorta was chosen, and the shape of the region could be changed in the evaluation. The findings of the present study demonstrated that the average noise of the LV in MBIR images was 51.9% and 30.2%, which is lower than in FBP and ASIR images, respectively. Similarly, the SNR of the LV in MBIR images was also found to be higher than in FBP and ASIR images. Similar results were also found in other tissues.

The present study was limited by the small sample size. In addition, different NI used in children with different age could also introduce bias. Further studies with larger sample sizes are warranted to confirm these findings.

In conclusion, images obtained from children and reconstructed with MBIR seemed more delicate and easy to review than FBP and ASIR images. These findings suggest that images reconstructed using MBIR were of higher quality and presented a lower noise compared with FBP and ASIR. We believe that images reconstructed with MBIR could still be used for disease diagnosis even after further reducing the radiation dose.
